# Computer based visualization of clot structures in extracorporeal membrane oxygenation and histological clot investigations for understanding thrombosis in membrane lungs

**DOI:** 10.3389/fmed.2024.1416319

**Published:** 2024-06-19

**Authors:** Maria S. Wagner, Michael Kranz, Lars Krenkel, Daniel Pointner, Maik Foltan, Matthias Lubnow, Karla Lehle

**Affiliations:** ^1^Department of Cardiothoracic Surgery, University Hospital Regensburg, Regensburg, Germany; ^2^Department of Biofluid Mechanics, Faculty of Mechanical Engineering, Technical University of Applied Sciences (OTH) Regensburg, Regensburg, Germany; ^3^Regensburg Center of Biomedical Engineering, Facility of University Regensburg and Technical University of Applied Sciences (OTH) Regensburg, Regensburg, Germany; ^4^Department of Internal Medicine II, University Hospital Regensburg, Regensburg, Germany

**Keywords:** ECMO, membrane lung, μCT, MDCT, shear induced clotting, vWF, histological evaluation

## Abstract

Extracorporeal membrane oxygenation (ECMO) was established as a treatment for severe cardiac or respiratory disease. Intra-device clot formation is a common risk. This is based on complex coagulation phenomena which are not yet sufficiently understood. The objective was the development and validation of a methodology to capture the key properties of clots deposed in membrane lungs (MLs), such as clot size, distribution, burden, and composition. One end-of-therapy PLS ML was examined. Clot detection was performed using multidetector computed tomography (MDCT), microcomputed tomography (μCT), and photography of fiber mats (fiber mat imaging, FMI). Histological staining was conducted for von Willebrand factor (vWF), platelets (CD42b, CD62P), fibrin, and nucleated cells (4′, 6-diamidino-2-phenylindole, DAPI). The three imaging methods showed similar clot distribution inside the ML. Independent of the imaging method, clot loading was detected predominantly in the inlet chamber of the ML. The μCT had the highest accuracy. However, it was more expensive and time consuming than MDCT or FMI. The MDCT detected the clots with low scanning time. Due to its lower resolution, it only showed clotted areas but not the exact shape of clot structures. FMI represented the simplest variant, requiring little effort and resources. FMI allowed clot localization and calculation of clot volume. Histological evaluation indicated omnipresent immunological deposits throughout the ML. Visually clot-free areas were covered with leukocytes and platelets forming platelet-leukocyte aggregates (PLAs). Cells were embedded in vWF cobwebs, while vWF fibers were negligible. In conclusion, the presented methodology allowed adequate clot identification and histological classification of possible thrombosis markers such as PLAs.

## 1 Introduction

Patients with severe cardiac or respiratory diseases are often treated with extracorporeal membrane oxygenation (ECMO) (1, 2). Despite the proven benefits for patients, this therapy is associated with hemostatic complications (3, 4). Clotting is the most occurring technical complication in ECMO (4, 5). Especially the ECMO membrane lung (ML) is a central point of clot formation (4). Within the ML, blood is spread across stacked fiber mats creating a high contact area for efficient gas exchange. This introduces two critical effects: Interaction of blood with foreign surfaces and non-physiological blood flow regimes. Large artificial surfaces trigger leukocyte adhesion and activation. This inflammatory response results in pro-thrombotic conditions within the ML (6–8). Furthermore, the ML design leads to a complex blood flow situation. It is known that blood flow has an essential influence on coagulation. Especially elevated shear rates introduce clotting effects by elongating the von Willebrand factor (vWF). Consequently, platelet aggregation and activation are initiated (9–11). These in turn might promote the clot formation in MLs. However, the complex blood flow conditions in ECMO aggravate to discern the exact localization of clot formation, potentially resulting in a spatial difference between visible thrombotic deposits and the region where clotting is initiated. Thus, it is not specified whether the ML is initiation point or effector point of clot formation. Further, the exact underlying interactions between the blood and the ML as well as the resulting clotting mechanisms are not yet sufficiently understood. Aim of this work was the development and validation of a procedure to capture key properties of ML clots, such as their localization, their size, the overall clot burden, as well as their composition. With this methodology, insights into the complex flow regimes and triggers for clot formation can be achieved.

## 2 Materials and methods

Thrombus extent and clot distribution were exemplarily analyzed within one clinically used permanent life support ML [PLS, Getinge/Maquet GmbH, Rastatt, Germany] using different imaging methods. Histological methods were applied to evaluate clot composition. The study design was prior approved by the Ethics Committee of the University Regensburg (vote no. 20-2051-104).

### 2.1 Technical details of the used system and patient data

The PLS ML was composed of polymethylpentene (PMP) gas exchange fibers and thermoplastic polyurethane (TPU) heat exchange fibers. It was divided into three sections: Blood coming from the centrifugal pump [Rotaflow, Getinge, Rastatt, Germany], entered the inlet chamber via a perforated plate (polycarbonate, PC), and passed (1) through a fiber mat stack (alternating gas (*n* = 23) and heat exchange (*n* = 22) fiber mats, perpendicular orientation), (2) the diving wall (DW, coarse PC-lattice acting as a support structure), and (3) a second fiber mat stack (74 gas exchange fiber mats, perpendicular orientation) from the outlet chamber to exit via another perforated plate. Total ML priming volume (MLPV) was 585 mL. The ECMO circuit was tip-to-tip coated with Bioline^®^.

The ML device reported in this study derived from a 54 year old male patient (Supplementary Table S1) who required venovenous ECMO (VV ECMO) due to Legionella pneumonia and sepsis. The technique of ECMO has been described previously (3). The patient was anticoagulated with Argatroban with an activated partial thromboplastin time (aPTT) of 50 ± 5 s (12). Inflammatory parameters improved within 5 days on ECMO. The patient required a system exchange on day 6 due to an acute ML thrombosis (pressure drop across the ML, dpML, increased from 20 to 46 mmHg) (13) accompanied by a worsening of gas transfer capability (decreased CO2 transfer, increase of gas flow). Gas transfer data improved after system exchange (4). The second ML (PLS, analyzed ML) was used for another 7 days. On day 11, weaning process was initiated with a successive reduction in blood flow and gas flow due to reduced requirement of ECMO support. Subsequently, platelet counts decreased (173 to 116 /nL), D-dimer levels increased (9 to 36 mg/L), and dpML/blood flow increased from 4.5 to 13.3 mmHg*min/l. On day 14, the patient was successfully weaned from the extracorporeal system.

### 2.2 Preparation of the ML

After termination of ECMO, the ML was rinsed extensively with 10 L isotonic saline to remove residual blood. The thrombotic deposits within the ML were fixed (paraformaldehyde, 4%, in 0.1 M phosphate buffer, pH 7.2 + 10% methanol, 1 L) and stored at 4°C (7, 14). For imaging, the fixative was drained and the ML was rinsed with 2 L isotonic saline followed by 2 L distilled water. The ML was dried with 1 L/min compressed air for 15 min. Metal parts on the outside of the ML (one screw and one spring) were removed to prevent scanning artifacts.

### 2.3 Imaging methods

**Figure 1:**
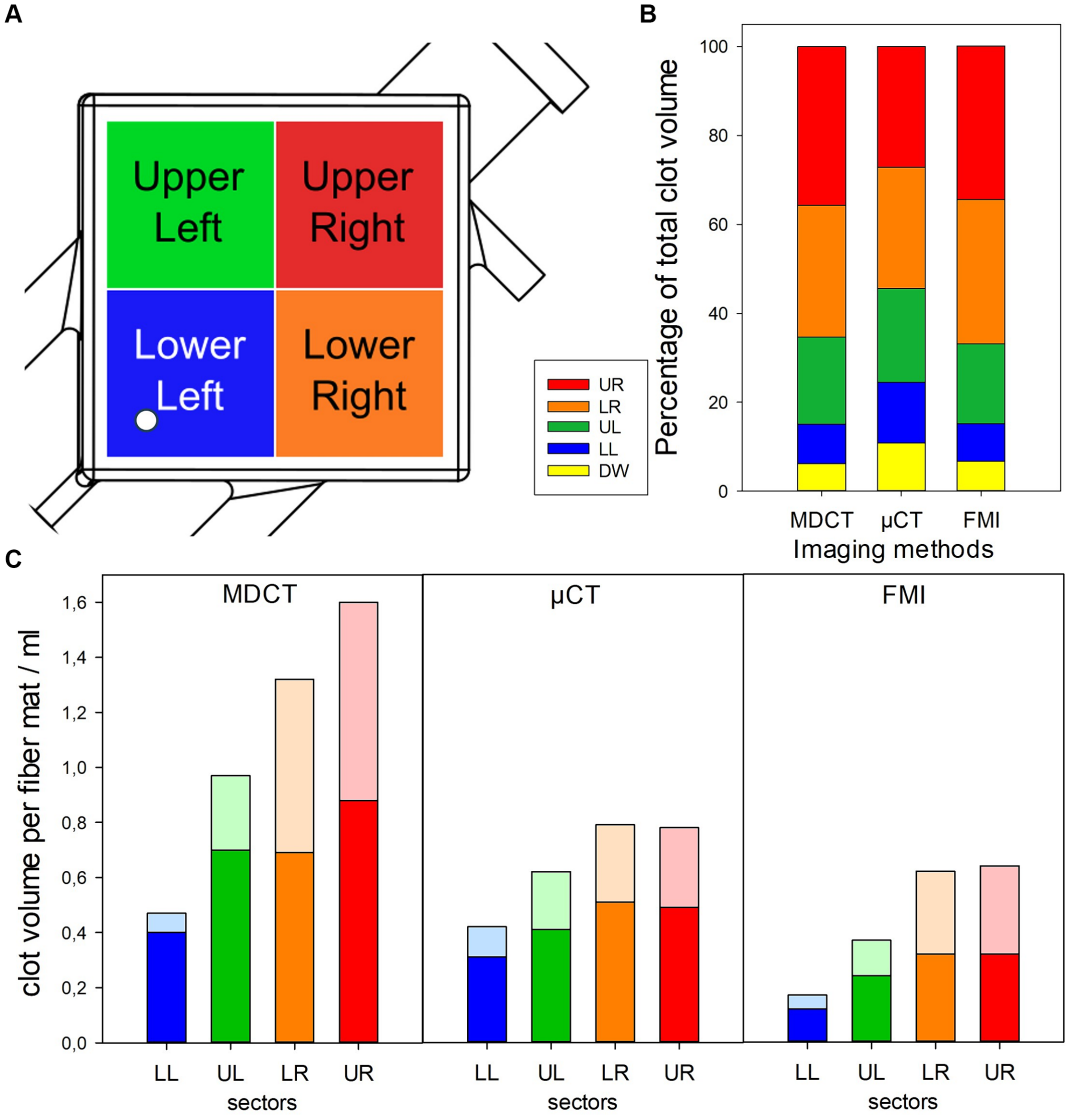
Visualizing of clot distribution in investigated ML using different imaging modalities. **(A)** Definition of investigated sectors of ML; white circle indicating position of inlet port. **(B)** Relative clot volumes in defined sectors. **(C)** Absolute clot volumes in defined sectors shown for inlet (opaque) and outlet chamber (transparent).

The ML was virtually divided into different regions of interest (ROIs). A square division in four sectors for inlet chamber and outlet chamber each was introduced [upper left (UL), upper right (UR), lower left (LL), and lower right (LR) sectors, Figure 1A].

#### 2.3.1 Multidetector computed tomography

**Figure 2:**
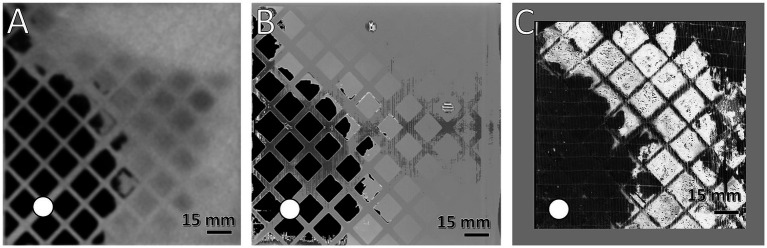
Comparison of the spatial resolution of used imaging methods. Visualization of clots at the DW using **(A)** MDCT, **(B)** μCT, and **(C)** first fiber mat after DW using FMI; clotted areas (bright) and clot-free areas (dark); (white circle) position of inlet port. Gray frame in **(C)** symbolizes framework to secure position of fiber mat during FMI, resulting in a smaller ROI.

**Figure 3:**
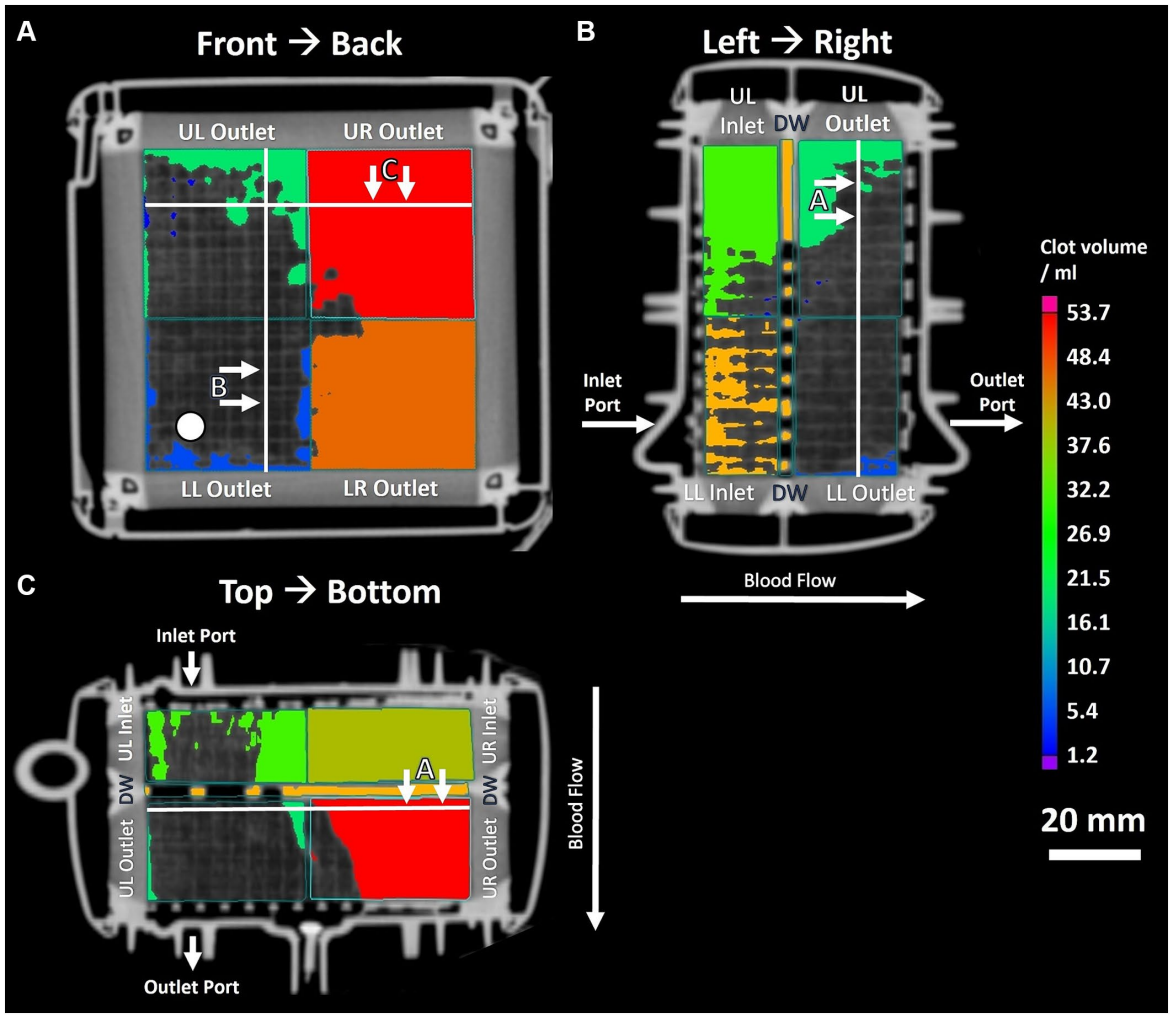
Three panel view of MDCT imaging showing clot distribution and clot volume within investigated ML. **(A)** Front to back view; white circle indicating position of inlet port. **(B)** Left to right view. **(C)** Top to bottom view. White lines indicating position of sectional views.

A Somatom Definition Flash MDCT [Siemens Healthineers, Erlangen, Germany] at the University Hospital Regensburg (software v.5.2.2) was used, based on ML scanning procedure provided by Dornia et al. with the “revised” MDCT settings by Birkenmaier et al. (1) and Dornia et al. (15) (Figure 2A). The empty ML was scanned with a scanning time of 5 s. Clot volume was calculated with VGStudio Max 2022.2 [Volume Graphics, Heidelberg, Germany] software by conducting a porosity analysis within the defined ROIs (Figure 3). The “Only threshold” algorithm was used with “Inclusion” set as analysis mode. Settings: minimal grayscale inclusion of −470.00, probability threshold 0.30, max. Diameter size 159.00 mm.

#### 2.3.2 Microcomputed tomography

**Figure 4:**
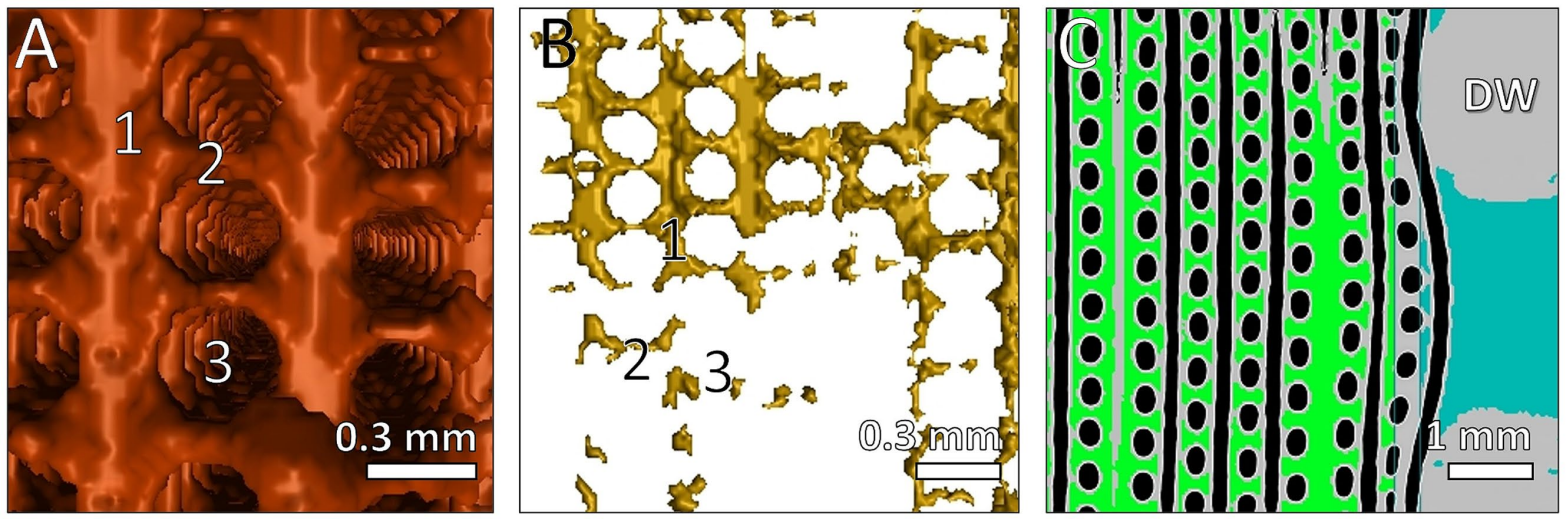
Reconstruction of clots structures between fibers visualized with μCT and VGStudio software. Fiber mats orientated in vertical direction. 3D reconstruction of detected clots with **(A)** high clot load and **(B)** low clot load. (1) 3D display of clots between fiber mats; (2) clots between two fibers within one mat; (3) location of membrane fiber. **(C)** 2D view of clots near the DW; 2D display of clots between fiber mats (green); clots within gaps of DW (blue); air inside membrane fibers (black); fibers and DW (gray).

The μCT settings as well as the procedure were based on Birkenmaier et al. (1). Briefly, the water connectors of the ML were cut off to fit inside the μCT. Imaging was carried out with a Phoenix v|tome|xs 240/180 μCT [Baker Hughes Digital Solutions, Hürth, Germany] with a scanning time of 40 h (Figure 2B). The clot volume calculation followed the MDCT procedure except the minimal grayscale inclusion of 0.0148. In addition, a 3D reconstruction of clot structures was performed (Figures 4A,B).

#### 2.3.3 Fiber mat image evaluation

**Figure 5:**
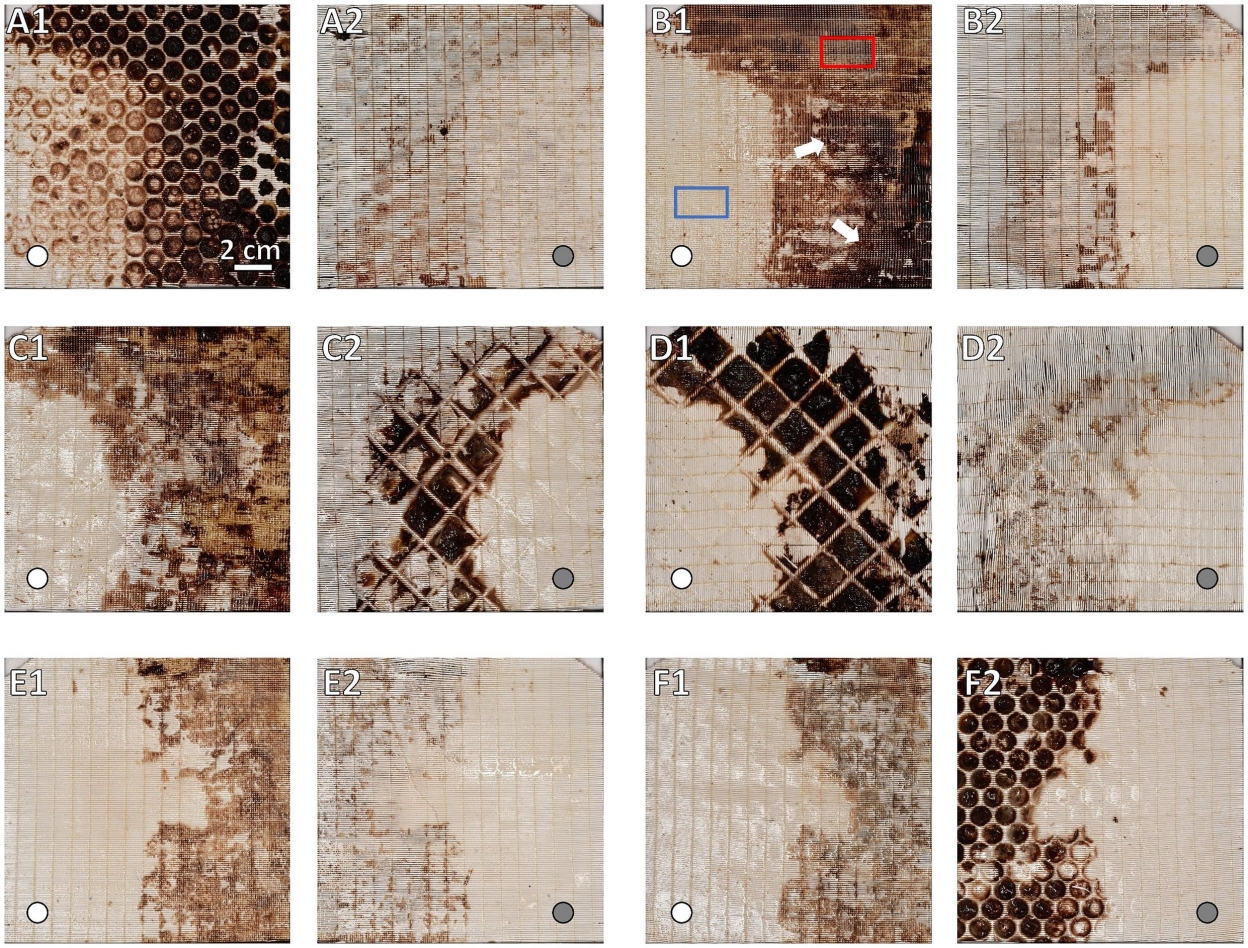
Macroscopic overview of clot burden on gas exchange fiber mats. Display of side facing the blood stream **(A1,B1,C1,E1,F1)** and their corresponding rear side **(A2,B2,C2,D2,E2,F2)**. Location of inlet port indicated by white circles in flow direction and gray circles opposite to flow direction. Gas exchange fiber mats **(A)** #1, **(B)** #9, and **(C)** #45 of the inlet chamber and fiber mats **(D)** #46, **(E)** #105, and **(F)** #119 of the outlet chamber. While in non-clotted regions the opaque fibers were visible, they were covered by brownish-red deposits in clotted areas. Darkness of the clots corresponded to clot thickness: Decrease in clot thickness along the course of the ML. The inlet chamber **(B1)** showing overall higher clot burden and clot thickness (darker clots) comparing to the outlet chamber **(E1)**. Clot burden predominantly in the upper left (UL), upper right (UR), and lower right (LR) sectors. A decrease in clot burden especially in the UL sector is visible. **(A)** First fiber mat after the first perforated plate; **(A1)** major clot burden in the interspace of the perforated plate; **(A2)** rear side of first fiber mat showing distinctly lower clot burden. **(B)** Representative gas exchange fiber mat of the inlet chamber; particularly dense clots indicated by white arrows; sample positions for histological investigation marked exemplarily: Visually clot-free area (blue), clotted area (red). **(C)** Fiber mat prior and **(D)** after the dividing wall (DW); **(C1)** similar clot distribution and burden compared to fiber mats of the inlet chamber (e.g., **B1**); **(C2,D1)** fiber mat sides adjacent to the DW; thick clots from the DW interspace were split during fiber mat retrieval; the imprint of the grid structure of the DW was visible; **(D2)** rear side of the first fiber mat of the outlet chamber with low clot burden. **(E)** Representative gas exchange fiber mat of the outlet chamber; clot distributed mostly in the UR and LR sectors. **(F)** Last fiber mat of the ML prior to the second perforated plate; **(F1)** similar clot distribution and burden compared to fiber mats of the outlet chamber (e.g., **E1**); **(F2)** again, major clot burden in the interspace of the grid of the second perforated plate.

For FMI, the ML was cut open and the fiber mats were removed from the stack. The mats were then clamped into a stabilization frame to facilitate photography (Figure 2C). Each side of all fiber mats (*n* = 119) was photographed (Figure 5). While thrombi appeared as red-brown (clotted) areas, gas permeable (clot-free) areas presented white color on the surface of the gas fiber mats (Figure 5). A computer algorithm converted the fiber mat images to binary images (threshold 160) and evaluated the area of black (clotted) and white (clot-free) pixels (Supplementary Figures S1A,B). The total clot volume was calculated from the sum of all clots of the individual fiber mat. Details of fiber mat preparation, technical details, instrument settings and processing of the image files see the Supplementary material section 1.2. After FMI processing, the ML was rehydrated in fixative (section 2.2) until further processing.

### 2.4 Histological investigation of fiber mats

Histological evaluation excluded heat exchange fiber mats (transparency of the TPU material). Only the opaque gas exchange fiber mats (PMP) were included in histological classification. Individual fiber mats were washed extensively with phosphate buffered saline (PBS). Samples (0.5 cm × 1.0 cm) from clotted and clot-free areas were prepared (Figure 5B1), stained, and visualized with a fluorescence microscope (section 2.4.1). Clotted areas (6 samples from randomly selected positions) presented multilayered deposits that prevented cellular evaluation (section 2.4.2). Visually clot-free areas from four gas fiber mats (two neighboring samples from each fiber mat), two from each ML chamber (inlet chamber, #9 and #13; outlet chamber, #105 and #119) from the LL sector (Figures 1A, 5B1) were analyzed. This sector was chosen due to the absence of clots across all fiber mats. Only the side of the sample facing the blood stream was examined. For further examination, the origin of the samples was blinded.

#### 2.4.1 Immunofluorescence staining and microscopic methods

Details of the staining protocol see Supplementary material section 1.3. Briefly, the two samples per fiber mat were stained with antibodies against CD42b/vWF, CD42b/CD62P, and fibrin. Fluorophore-conjugated secondary antibodies were used for visualization. Finally, samples were placed between two cover slips and embedded in Fluoromount-G^®^ + DAPI [ThermoFisher, Waltham, United States]. Imaging was performed using a fluorescence microscope [Leica DMRBE, Leica Microsystems, Wetzlar, Germany] and a Spot2000 camera [Diagnostic Instruments, Sterling Heights, MI, United States] under software control [Visiview^®^, Visitron, Puchheim, Germany].

#### 2.4.2 Histological image evaluation

**Figure 6:**
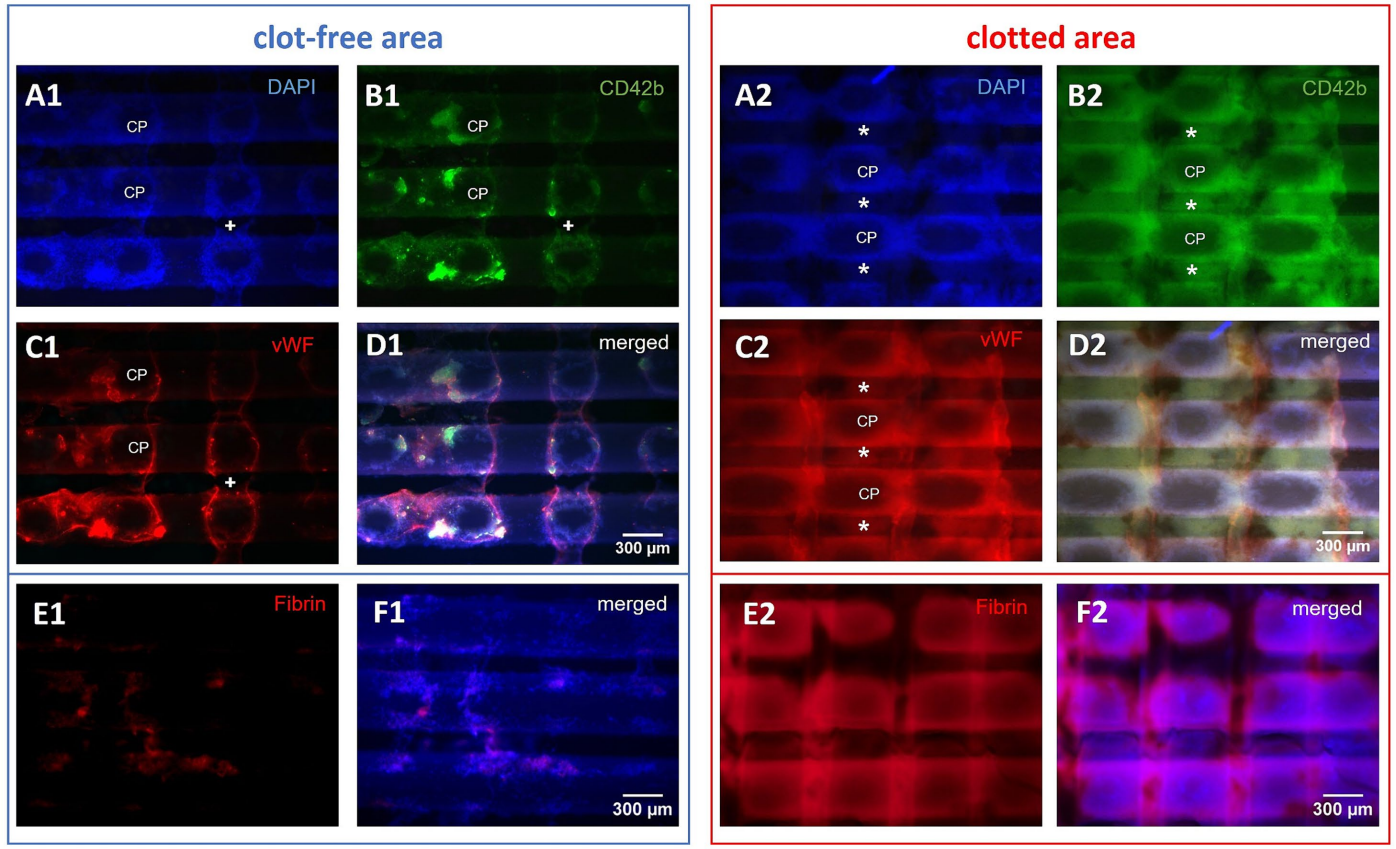
Microscopic top views of a gas exchange fiber mat. Investigation of the side facing the oncoming blood stream. Representative microscopic top views of three horizontal gas exchange fibers of clotted and clot-free areas, displaying nine crossing points (CP): **(A1–F1)** In the visually clot-free areas single cell adhesion to fibers was predominant; thus, this region was accessible for qualitative and quantitative evaluation; occasionally bridging deposits (+) spanned the interfiber space. **(A2–F2)** Clotted regions consisted of multi-layered deposits building up around CPs connecting neighboring fibers via pseudomembranes (*); multi-layers limited the investigation to quantitative description. **(D)** The merged micrographs showed congruent deposits consisting of **(A)** DAPI-stained nucleated cells, **(B)** platelets, and **(C)** vWF; **(E)** fibrin and **(F)** fibrin merged with DAPI.

Due to the overlay of multiple cell layers, clotted areas (Figure 5) were analyzed only qualitatively (25- and 50-fold magnification). Immunofluorescence (CD42b/vWF, CD42b/CD62P, fibrin, and DAPI) was used to characterize the structure of the deposits as well as the co-localized compounds (Figures 6A2–D2). Of particular interest were the crossing points (CP) of adjacent gas fibers (*n* = 18; 50-fold magnification).

**Figure 7:**
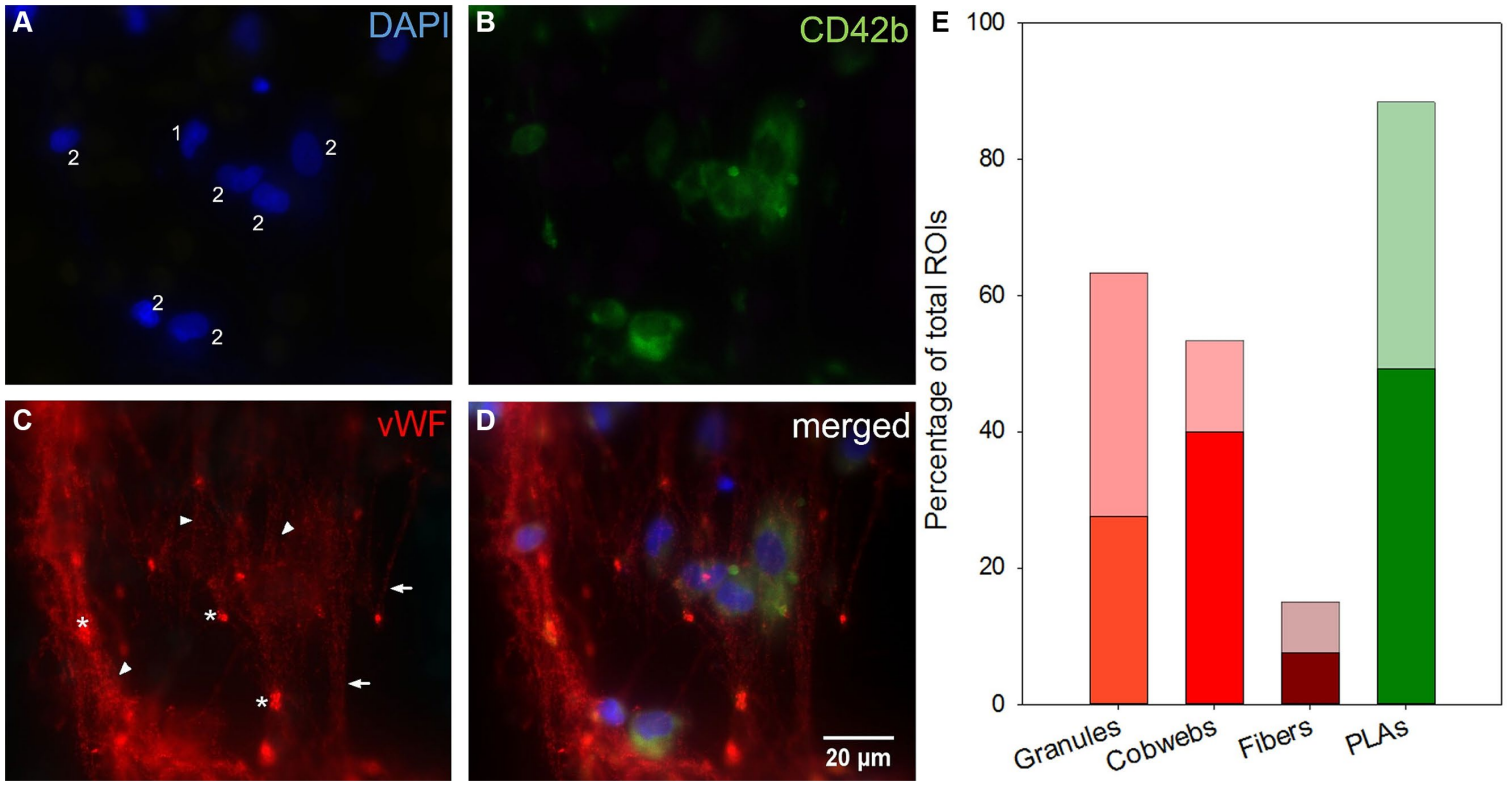
Microscopic top view of deposits within a clot-free area and their distribution along the fiber mats. **(A)** Leukocyte nuclei in DAPI-staining. **(B)** Platelets stained against CD42b. **(C)** Different vWF structures: VWF fibers parallel aligned (arrows) or linked as cobwebs (arrowheads) and vWF granula (*). **(D)** Merging images **(A–C)** allowed the co-localization of structures: Nucleated cells were associated with platelets as platelet leukocyte aggregates (PLAs, 2) or entangled in the vWF-cobwebs (vWF nuclei, 1 and 2) without (1) or with platelet adhesion (vWF-PLAs, 2). **(E)** Prevalence of vWF granules, cobwebs, and fibers and PLAs expressed as the percentage of total ROIs in inlet (opaque) and outlet chamber (transparent).

**Figure 8:**
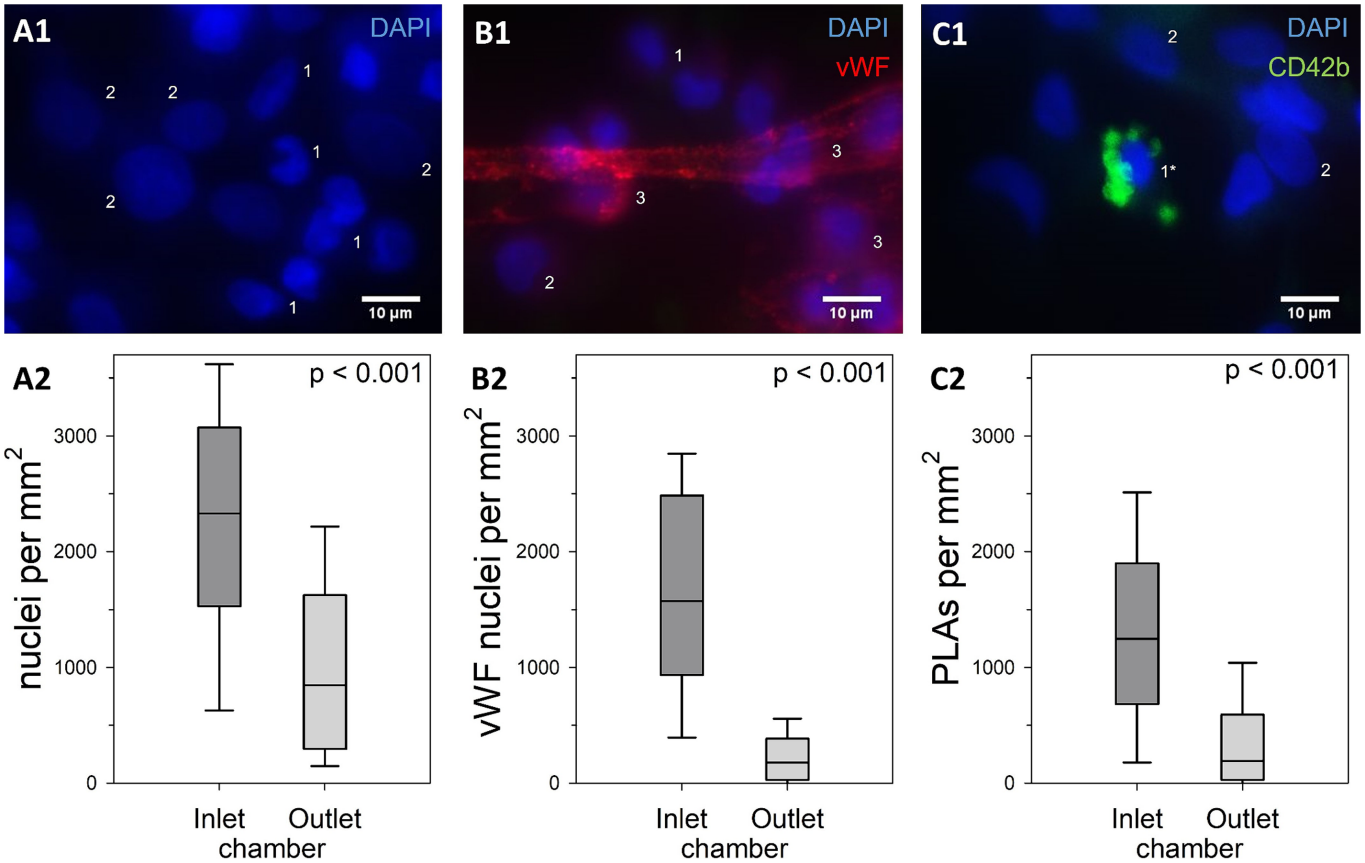
Comparison of cell densities within clot-free areas of the inlet and the outlet chamber. **(A1)** DAPI-stained nuclei with (DN, 2) or without (1) decondensed nuclear structure. **(B1)** Attachment to any vWF structure qualified these nuclei as vWF nuclei (3). **(C1)** CD42b-positive platelets attached to a nucleus defined a platelet leukocyte aggregate (PLA, 1*). Cell densities of the main categories **(A2)** total nucleated cell count, **(B2)** vWF nuclei, **(C2)** and nuclei part of PLAs; results of the two ML chambers were compared using Mann–Whitney U tests with shown *p*-values; inlet chambers displayed significantly higher nuclear densities than outlet chambers in all categories.

Clot-free areas (Figure 5) allowed both, qualitative and quantitative evaluations. Overviews (25-fold magnification) were used to estimate the overall cell density (low, medium, high), the extent of cells around CPs (none, low, strong), and the prevalence of small fiber-bridging deposits (Figures 6A1–D1) (7). In samples with CD42b/vWF-staining each ROI was investigated regarding the prevalence of different vWF structures according to Steiger et al. (16). Table 1 includes definitions of vWF-positive fibers, cobwebs and granules. Representative photographs of vWF fibers, cobwebs and granules are displayed in Figure 7. Each of the structures was rated as prevalent or missing in each ROI. In the same way the presence of platelet leukocyte aggregates (PLAs) was described (Figure 7). For quantitative analysis, 30 randomly selected positions per sample were photographed (400-fold magnification, warp threads excluded) and the density of DAPI-stained nuclei was determined using the cell counter function of ImageJ 1.49u [National Institutes of Health, Bethesda, United States]. Used ROIs were defined as the part of the gas exchange fiber horizontal to the microscopic level (1,600 × 600 pixels) excluding cells on the rounded edges of the hollow fibers. Thus, an area of 1.011 mm^2^ was evaluated per specimen. Specific staining properties defined different cell categories (Table 1). A subpopulation of the nuclei was identified as swollen and decondensed nuclei (DN) with enlarged, rounded or egg-shaped nuclei (Figure 8A1). These cells were also subdivided in subcategories as described in Table 1. To determine the size of DNs, 100 DNs were randomly identified and measured (length: maximum Feret’s diameter; width: perpendicular on the center of the length). Their cross-sectional surface was approximated as an ellipse with A_ellipse_ = 0.25 π * length * width. Representative histological stainings were presented in Figure 8A1 (DN), Figure 8B1 (vWF-nuclei), Figure 8C1 (PLAs), and Supplementary Figure S2 (CD62P-PLA).

**Table 1:** Identification of different histological categories of cells and vWF structures per ROI.

Categories	Staining properties	Description
Total nuclei	DAPI+	All DAPI-stained nuclei
vWF nuclei	DAPI+/vWF+	DAPI-stained nuclei attached to any of the vWF structures
PLAs	DAPI+/CD42b+	DAPI-stained nuclei with attached CD42b-positive platelets
CD62P-PLAs	DAPI+/CD42b+/CD62P+	DAPI-stained nuclei with attached CD42b- and CD62P-positive platelets
vWF-PLAs	DAPI+/CD42b+/vWF+	DAPI-stained nuclei with attached CD42b- positive platelets and vWF structures
vWF fibers	vWF+	Rarely cross-linked vWF threads (single, non-parallel or parallel orientated threads; thicker fiber bundles)
vWF cobwebs	vWF+	Frequently cross-linked vWF threads, cobweb structures (distance between cross-links <10 μm) with or without attached cells
vWF granules	vWF+	Spot-like vWF deposits (intra- and extracellular granules)

### 2.5 Data processing and statistical methods

The image processing algorithm was established using PyCharm 2022.3.2 Professional [JetBrains, Prague, Czech Republic] based on Python 3.10.9 [Python Foundation, Delaware, United States]. SigmaPlot 13.0 [Systat Software, Inc. San Jose, United States] was used for statistical analysis. Non-gaussian distribution (all results): Wilcoxon test to check differences between inlet chambers and outlet chambers of all investigated sectors of the ML when using FMI; due to the different geometrical sizes of the chambers, a different sample size was given; fiber mat images adjacent to the DW were not included in the calculation because they did not depict clots of either the inlet or outlet chamber, but clots of the DW. In order to perform the Wilcoxon test, the 89 remaining inlet fiber mat images and the first 89 outlet fiber mat images were taken into account. For comparison of the sectors Mann–Whitney U test was used. To compare histological results of the two ML chambers were tested by Mann–Whitney U. The number of nuclei in each ROI was extrapolated to an area of 1 mm^2^ prior to defining the density of nucleated cells per ML chamber. Data was presented as median (interquartile range, IQR). For all tests a level of significance α = 0.05 applied.

## 3 Results

### 3.1 Clot volume and distribution

All three imaging methods showed a different total clot volume. MDCT calculated the largest clot volume (274 mL, 48% MLPV). The inlet chamber, the DW and the outlet chamber contained 120 mL (21% MLPV), 16 mL (3% MLPV) and 138 mL (24% MLPV), respectively. The μCT imaging method resulted in a total clot volume of 160 mL (27% MLPV) with 78 mL (13% MLPV) in the inlet chamber, 17 mL (3% MLPV) in the DW, and 65 mL (11% MLPV) in the outlet chamber. FMI showed the smallest total clot volume of 113 mL (20% MLPV) with 46 mL (8% MLPV) in the inlet chamber, 8 mL (1% MLPV) in the DW, and 59 mL (10% MLPV) in the outlet chamber. The UR and the LR sectors indicated significantly higher clot volumes than UL and LL sectors independent of the imaging method (Figure 1B). While the lowest clot volumes were detected in the LL sector, the highest clot volumes were calculated in the UR sector (MDCT, 36–93 mL; μCT, 22–44 mL; FMI, 9–39 mL). Finally, independent of the imaging method the mean clot volume per fiber mat was significantly higher in the inlet chamber compared to the outlet chamber (Figure 1C).

The imaging methods differed in their spatial resolution due to their process of image acquisition, exemplarily displayed at a representative position at the DW in Figure 2. All imaging modalities showed a clot-free area (dark) in the LL sector and a clotted area (bright) in the other sectors. However, while MDCT and μCT presented a continuous clot in these sectors, FMI showed a clot-free area in the UR sector (Figure 2C). Figure 3 presents clot analysis using MDCT and depicts the spatial distribution of the clots at different levels within the ML. The clot within the UR and LR sectors penetrated the whole ML in flow direction (Figure 3). Furthermore, a widening of the clot-free areas in the left sectors from inlet to outlet chamber (lateral view and top view) was detected.

μCT scanning and 3D-reconstruction (VGStudio software) of ML clots demonstrated the highest spatial resolution that allowed presentation of details of clot deposits on the individual gas fiber (Figures 2, 4). As shown in Figures 4A,B, clot structures were mainly located between the fiber mat layers. Clot formation spanned across neighboring fiber mats through the gaps between the membrane fibers. Furthermore, μCT approved the heterogeneous distribution of the fiber mats (Figure 4C) in the inlet chamber before the DW with complete clot filling of blood-bearing spaces (green). A displacement of the fiber mats in blood flow direction became evident especially in proximity of the DW (blue).

FMI was capable of indicating qualitative differences in the thickness of clot structures. Thicker clots appeared darker and were found predominantly in the inlet chamber (Figures 5B1,C1) while clots of the outlet chamber were thinner and thus appeared brighter in the FMI images (Figures 5E1,F1). Moreover, FMI highlighted irregular clot burden on a few special fiber mats more explicitly than MDCT or μCT: The first and last fiber mat of the ML (Figures 5A1,F2) as well as the fiber mats adjacent to the DW (Figures 5C2,D1). Thick clots deposed in the interspace of the two perforated plates and the DW. However, all other fiber mats showed similar clot distribution. Clots were found in the UL, UR, and LR sectors mainly attached to the side of the fiber mat facing the blood stream. In contrast, the rear side showed only a few clots attached. Over all, a relatively constant volume of clot deposits in the UR and LR sectors in both chambers (Supplementary Figure S1C, red and orange curves) was detected. Both sectors showed comparable clot volumes per fiber mat with a slightly decrease along the fiber mats of the outlet chamber (*p* = 0.072). The LL sector contained the smallest clot volume per fiber mat with significantly lower levels in the outlet compared to the inlet chamber (Supplementary Figure S1C, blue curve, *p* < 0.001). The UL sector showed a significantly higher clot volume (Supplementary Figure S1C, green curve) than the LL (*p* < 0.001) and a significant smaller clot volume than the UR (*p* < 0.001) and LR (*p* < 0.001) sector. The overall clot volume per fiber mat of the UL sector was significantly higher in the inlet compared to the outlet chamber (*p* < 0.001).

### 3.2 Histological investigation—preliminary analysis of thrombogenic markers

Based on the spatial distribution of clots within the analyzed ML, this study used histological antibody staining to demonstrate the extent of cellular colonization and to identify different cell populations and vWF structures in clotted as well as clot-free areas in the inlet and outlet chambers of the ML. This was only a preliminary study.

#### 3.2.1 Fiber-spanning multi-layers of leukocytes, platelets, fibrin, and vWF accumulations in clotted areas

Clotted areas of the fiber mats (Figures 6A2–D2) consisted of multi-layered deposits spanning neighboring gas fibers [pseudomembranes (7)]. Single cell analysis failed due to the opaque nature of the deposit structure. The deposits included large amounts of nucleated cells and cell aggregates (Figure 6A2) as well as extended accumulations of CD42b-positive platelets (Figure 6B2), vWF-positive structures (Figure 6C2), and fibrin (Figure 6E2). All of these compounds were located in congruent areas (Figures 6D2,F2). Leukocytes, platelets and vWF mainly accumulated around the CPs. Only the immediate centers of CPs were omitted showing the naïve fibers with the outward building up deposits creating a 3D grid-like structure. In 25-fold magnification the apparent surface of the deposits displayed linear elevations perpendicular to the fibers of the specimen. Between the elevations U-shaped gaps bearing the CPs in their center were created as the remnants of the adjacent fiber mat. These deposits were mostly found on the side of the sample facing the oncoming blood stream. The rear side of the specimen was visually white and microscopically resembled the naïve gas exchange fibers with only very limited adherent nucleated cells (data not shown).

#### 3.2.2 Association of adherent leukocytes with PLAs and vWF granules and cobwebs in clot-free areas

Almost all investigated specimens presented vWF in form of granules, fibers or cobwebs (Figure 7). Granules were the most frequently recorded vWF structure in ROIs [57 (44–89) %]. They were either entangled in cobwebs often located at the branching points within the nets or attached to vWF fibers or located in close proximity to nucleated cells. Cobwebs appeared with a frequency of 52 (23–85) % and vWF fibers were rarely detected [15 (10–20) %]. DAPI-stained nuclei were often attached to fibers and cobwebs. Both granules and fibers were evenly distributed throughout the ML. Cobwebs dominated in the first fiber mats of the inlet chamber. Furthermore, PLAs were omnipresent and occurred in 93 (74–98) % of ROIs per sample.

The clot-free areas of the gas exchange fibers were covered with a continuous and fiber encompassing cell layer (Figure 6A1; Table 2). In the outlet chamber, the cells were more evenly but less densely distributed along the gas fibers compared to the inlet chamber. The cells accumulated around the CPs with the highest densities on gas fibers from the inlet chamber. All samples contained small fiber-bridging membranous deposits (no extended pseudomembranes). Fibrin deposition to the surface of fibers was merely found in visually clot-free areas, only in areas of aggregated DAPI-stained nuclei (Figures 6E1–F1).

**Table 2:** Appraisal of the leukocyte coverage on clot-free specimen from inlet and outlet chambers.

	Inlet chamber	Outlet chamber
Amount of samples	4	4
Overall cell density (low/medium/high) (%)	0/25/**75**	50/50/0
Cell density around CPs (low/medium/high) (%)	0/0/**100**	50/50/0
Fiber-bridging membranous deposits (%)	100	100

Bold numbers indicate maximum values.

To quantify cell density on the gas fibers, more than 13,400 nucleated cells were evaluated from 8 specimens with a total ROI of 8.1 mm^2^. Cells with normal and decondensed nuclei (Figure 8A1) as well as different subpopulations (Table 1; Figures 8A2; Supplementary Figure S2) were quantified.

The median total cell density was 1,573 (579–2,522) nuclei/mm^2^ with significantly higher cell densities in the inlet compared to the outlet chamber (*p* < 0.001). About 10% of the nuclei were identified as DN [inlet: 11 (7–16) %; outlet: 8 (2–16) %; *p* = 0.024]. DN displayed a median length of 13 (12–15) μm (maximum Feret’s diameter) and a median width of 9 (8–11) μm with an approximated cross-sectional elliptic surface area of 97 (73–120) μm^2^.

VWF nuclei appeared with a density of 504 (156–1,573) cells/mm^2^ [45 (19–71) % of total nuclei] and were more prevalent in the inlet than the outlet chamber (*p* < 0.001) (Figure 8B2). The majority of these cells [64 (35–90) %] was co-localized with platelets (vWF-PLAs). The median density of PLAs was 623 (119–1,335) /mm^2^ (41 (20–67) % of total cell count). PLAs were more than twice as prevalent in the inlet compared to the outlet chamber (*p* < 0.001) (Figure 8C2) and accounted for 56 (38–77) % and 28 (6–45) % of nucleated cells, respectively (*p* < 0.001). The majority of PLAs presented the activation marker P-selectin [CD62P-PLAs, 75 (61–91) % of PLAs; inlet: 79 (65–92) %; outlet: 66 (54–84) %; *p* = 0.042].

## 4 Discussion

This study critically compared three imaging methods to localize clots and quantify the clot burden inside a ML. The methods differ in their measurement sensitivity but also in the complexity of the mechanical equipment and the evaluation algorithm. While μCT featured by high resolution at the membrane level, FMI and MDCT allowed adequate localization of clots, but without precise delineation. The quality of MDCT was sufficient to visualize the extended clot burden as well as clot-free areas in the ML. CT devices are very expensive and only available in special institutes, while FMI only requires a camera and freely available evaluation software. CT measurements are carried out in the naïve ML while FMI requires the ML to be opened and the gas fiber mats to be mechanically separated from each other. However, the latter allowed histological examinations at defined locations in the ML. Specific structures were identified including fiber-spanning pseudomembranes consisting of leukocytes, vWF, fibrin, and platelets in clotted areas as well as extended cellular coverage with leukocytes and PLAs, embedded in vWF cobwebs in clot-free areas.

### 4.1 Advantages and disadvantages of the imaging methods to localize clots and estimate clot volume

The three imaging methods showed a similar clot distribution within the investigated ML. However, the calculated clot volumes differed significantly.

MDCT showed the highest total clot volume. This resulted from the relatively low spatial resolution of MDCT which did not allow to accurately distinguish between membrane fibers and clots. Consequently, more structures were assumed to be clots than existed. In this study, clots were detected with MDCT following the protocol previously described by Birkenmaier et al. (1). Dornia et al. published the approach of detecting clot formation within MLs using MDCT in 2013 (15, 17, 18). Thus, the chosen standard settings showed some limitations—not all clots were detected by Dornia’s protocol due to the different attenuation coefficients. Therefore, Birkenmaier et al. provided “revised” MDCT settings with an attenuation display at the center of −280 HU (Hounsfield unit) and a width of 560 HU (1). These settings were used in our study, and a sufficient detection of clots was possible which confirmed Birkenmaier’s method. Thrombotic structures were detected over the entire ML geometry including ML housing and tube connectors. Clot structures were not manipulated during the imaging process as no dismantling of the ML housing was necessary. The short scan time of 5 s, low preparation time, and wide availability of MDCTs in clinical facilities make MDCT a suitable technique for visualization of clots in MLs. Clinically used MDCT devices are designed to use a relatively low radiation dose which is sufficient for patients but results in a lower spatial resolution. Structures up to a size of 0.5 mm are displayed properly. Thus, the exact shape of the clots or membrane fibers could not be described adequately and the clot volume appeared larger than in reality. However, clots could be localized in certain areas within the ML.

In contrast, high spatial resolution (50 μm) of industrial μCT even permitted the detection of small deposits between the fiber mats and between individual fibers as well as large clot structures in all areas of the ML. The higher spatial resolution derived from a significantly higher radiation dosage in comparison to the MDCT. In contrast to the applications of μCT according to Birkenmaier et al. (1), μCT is used less for medical issues and more in materials science to analyze cracks and pores on certain material surfaces. In the present study, μCT allowed highly detailed information on the shape of small and large clots within MLs. Analogous to MDCT, no dissection of the ML was required for μCT either, so that the clot structures remained virgin and detailed examinations are possible. For example, a displacement of the fiber mats within the inlet chamber near the DW was detectable with μCT (Figure 4C). In previous μCT scans of a naïve ML (unpublished data) a fiber mat shift was also detectable. It remained unclear whether the shift of the fiber mats increased during the ECMO run in addition to the initial shift. Furthermore, μCT proved the heterogeneous arrangement of the individual gas fibers within the ML. This manufacturing limitation aggravates an accurately prediction of local shear forces. A disadvantage of this method is the long scanning time (about 40 h) to distinguish between membrane fibers and clot structures. In addition, a long post-processing and evaluation time (about 10 h) is required. This implicated higher costs per ML compared to MDCT. With this procedure, higher acquisition costs for the device and trained personnel must be planned for, while MDCT is routinely used in hospitals. Furthermore, both CT imaging methods required a drained ML (removed from patient’s circulation). It was important that the clot-free areas were filled with air to adequately distinguish between clotted and clot-free (air) areas within the ML. This means, CT scans of MLs during ECMO operation cannot detect clots with this method.

μCT calculated by a factor of 1.7 less clot volume of the analyzed ML than MDCT. Birkenmaier et al. showed similar differences with high clot loadings, while MLs with little to no thrombotic deposits resulted in a comparable clot burden due to higher resolution of the μCT (1). Panigada et al. identified that clots deposited more frequently in the inlet chamber and the UR corner which both can be seen in our results (19). Fu et al. conducted numerical investigations of different MLs (20). They investigated the accumulated residence time (ART) inside a similar ML. Their results showed an uneven distribution of blood flow and a high ART in the UR corner and the outlet chamber (20). They expected an elevated risk of thrombosis in those areas of the ML due to blood stasis (20). However, Dornia et al. and Birkenmaier et al. reported that clots were predominantly in the lower parts of ML (1, 15). While in the present study 60% of the total clot volume was found in the inlet chamber, Birkenmaier et al. found clot loadings of 36, 74, and 55% in the inlet chamber (1). Our observation that more thrombi were present in the inlet chamber than in the outlet chamber does not seem to be a general statement. The heterogeneous distribution could be due to the patients underlying disease, anticoagulation or duration of the system. Due to the heterogeneity of clot formation in the MLs mentioned, it is advisable to investigate further MLs.

In contrast to MDCT and μCT, FMI was a simple and inexpensive method to localize clot structures very accurately within the ML. However, it only processed 2D images. To obtain volume information, mathematical assumptions and approximations were necessary to develop the algorithm. Furthermore, the ROI was smaller than the other methods due to the photography frame required. This frame concealed the edge areas of the fiber mats. By this FMI provided the lowest clot volume in the ML as it did not detect frame covered clot structures. Moreover, FMI required a mechanical separation of the fiber mats which resulted in a division of clots. It was observed that during the removal of the fiber mats, the clots mainly remained on the side of the subsequent fiber mat facing the blood flow or partially within the DW (Figure 2C, upper right area; Figures 5C2,D1). This should be considered as the clots deposed predominantly in the space between the fiber mats (Figures 4A,B). As a result, the destruction of the clot integrity may give incorrect information on the clot volume and shape. Thus, FMI indicated different clot layer thicknesses due to brightness appearing in the images (Figure 5). The entire imaging and evaluation process took about 6 h for one person. The fiber mats had to be removed from the ML for photography. This required the utmost care to avoid cutting or damaging the clots. However, this method did not allow the investigation of blood clots in tube connectors or in the housing. Additionally, only deposits with a different color were detected reliably. Despite these limitations, the distribution of clots in the ML was comparable to the other imaging methods. In addition, the intensity of the red color provided information about the compactness of the associated thrombus and can be tracked across different levels in the ML (in the inlet chamber). Therefore, FMI represents an attractive method that can be used by everyone to locate clots in a ML and classify them histologically.

In summary, the locations of clotted and clot-free areas could be accurately determined with all three imaging methods. However, the underlying flow conditions in the ML are highly complex. Isolated clot detection is not sufficient to investigate flow phenomena. Histological classification of the identified clots or clot-free areas seems to be a promising way to identify mechanism of clot formation including flow-induced thrombosis (vWF). In the present study, imaging suggested that a variety of immunological reactions might take place especially in the inlet chamber due to the high clot load.

### 4.2 Inflammatory responses, PLAs, and decondensed nuclei as trigger factors for thrombus formation within MLs

Histological clot classification and identification of functional molecules and blood cells (vWF, fibrin, platelets, leukocytes, and PLAs) are important to gain mechanistic insights into thrombus formation (21) of thromboembolic diseases like acute ischemic stroke (22) or during ECMO therapy (23, 24).

Visual inspection of the gas fiber mats using FMI identified large areas of red thrombi. These appeared in histological analysis as fiber-spanning multi-layers including leukocytes, platelets, vWF, and fibrin accumulations. The presence of both vWF and fibrin suggested that stable thrombi were formed in clotted areas. This may be a response of the thrombin-catalyzed conversion of the plasma protein fibrinogen to fibrin (25). However, this reaction did not occur in the initial stage in contact with the naïve gas exchange surface in clot-free areas. These areas were covered with more or less evenly distributed leukocytes, platelets and PLAs that were incorporated in vWF cobwebs.

The heterogeneous distribution of the clots may be a result of different flow conditions within the ML. Both, areas with high shear rates (at the immediate entrance of the PLS ML) (1, 26) and stagnation points (27) (reduced blood flow attributed to the weaning process) are discussed. However, this mechanism is questionable, since clot localization within other used PLS MLs after weaning presented various distribution patterns (1). A reasonable explanation for the non-uniform clot formation could be the irregular arrangement of the gas fibers, which complicates the simulation of local shear forces within the ML.

To avoid this problem, vWF—an indicator for shear-induced clot formation (28–38)—was visualized within clotted and clot-free areas. Pathological flow conditions caused unfolding of the compact vWF molecule and formed elongated fibers orientated in flow direction that could assemble into fiber bundles or cobwebs (31, 39, 40). Both morphologies were part of the thrombotic deposits on gas exchange fibers from MLs of 21 ECMO patients (16). However, existence of vWF-fibers (29%) or cobwebs (43%) was low and did not correlate with clinical or technical data. This may be due to the random selection of the samples without assignment to the localization within the ML. In the present study, vWF dominated in the clotted areas. However, it was impossible to identify different vWF structures within the multi-layered pseudomembranes. These structures prevented a quantitative clot analysis. To maintain the original clot structure and allow insights into these structures, serial cross-sections of tissue-embedded ML samples would be required. These failed up to now due to air-filled gas fibers that disintegrated the sections.

While clotted areas represent the advanced product of the coagulation cascade, the clot-free areas reflect the initial response of blood with the artificial surface and local shear forces. These surfaces were covered with nucleated cells partially associated with vWF cobwebs and platelets (PLAs). This was in agreement with previous studies (3, 7, 14, 16). Contact of blood with artificial surfaces after initiation of ECMO triggers complex inflammatory responses mediated by interdependent cellular and humoral activation pathways (8, 41) similar to that seen in systemic inflammatory response syndrome (SIRS) (42). As a result, CD45-positive leukocytes and mesenchymal-like cells (CD90+/CD105+) adhered onto the ECMO surfaces (6, 7). Co-localized vWF fibers were only partially detectable (<20% of ROIs) and are therefore questionable indicators of shear-induced thrombus formation. In contrast, omnidirectional cobwebs with integrated platelets and nuclei may also be a product of shear induced conformational changes of the vWF (39). However, the preparation process prevented a clear statement about the orientation of the vWF fibers/cobwebs in flow direction. Of particular interest was the dominance of PLAs [64 (35–90) %]. Almost all PLAs were P-selectin-positive [75 (61–91) %]. In contrast, Steiger et al. identified only one third of adherent cells on ML fibers as PLAs that were defined as vWF granules in close proximity to nuclei (vWF nuclei) since vWF was stored as granular structures in α-granules of platelets (16, 43). In the present study, the discrimination of vWF granules as intra- or extracellular (16) was not carried out because high vWF prevalence especially within vWF clots or cobwebs masked the presence of granules. The differences in the frequency of vWF nuclei were probably due to the fact that Steiger et al. analyzed randomly selected samples of multiple MLs, which might also explain different PLA levels (16).

PLAs obviously play an important role in clot formation (29, 30, 44). PLAs were not only part of clots within MLs but were also found in blood from ECMO patients (24, 45). However, there was no correlation with thromboembolic events during ECMO therapy. Nevertheless, there was a link between PLAs, platelet activation, blood coagulation and thrombus formation in patients in the very early phase of acute myocardial infarction (AMI) (46). PLAs were detected in blood from patients with acute coronary syndrome (ACS) as well as in ruptured plaques from patients with AMI (47, 48). Furthermore, patients with COVID-19 had significantly elevated levels of circulating cellular clusters (PLAs as well as circulating leukocyte clusters) that correlated with thrombotic complications (49). In summary, high clot burden in the inlet chamber correlated with a higher dominance of adherent leukocytes and PLAs compared to the outlet chamber.

In the literature, two opposing theories for the formation of clots in the ML are discussed: First, the ML might act like a sieve. The stacked fiber mats of the ML could trap clots that have previously formed in the extracorporeal circuit, suggesting that the ML is the effector point but not the trigger for thrombogenesis (26, 50). Second, changes in blood flow and shear rates upon entry into the ML could activate clotting, leading to clot formation in the ML itself (51, 52). Both theories supported the results that clots mainly formed in the vicinity of the blood entrance of the ML. Contrary to Hastings et al. where only loosely attached thrombus material was found in the pre-membrane perforated plate (50), thrombotic deposits were found continuously throughout the investigated ML indicating that clots originated within the ML. Our findings supported that a combination of different factors triggered clot formation within an ML: Shear induced thrombus formation might have led the process especially in the inlet chamber and along the direct blood stream axis while low to no flow regimes along the upper ML edges might have resulted in visually red clots. The thrombo-inflammatory response of immune-active cells to the artificial surface might have created a base for pro-thrombotic conditions throughout the entire ML.

### 4.3 Limitations

Our study proved the feasibility of the developed methodology. Therefore, only one ML was analyzed. Histological examination of one ML only provides an indication of which mechanisms are involved in clot formation. The histological analysis was restricted to vWF structures, leukocytes, and PLAs. Other mediators would be conceivable (e.g., detection of neutrophil extracellular traps). One critical limitation of the histological processing was the preparation of the samples (top view), which would not allow any statement on changes of cellular alterations along the blood stream. To obtain the original deposits cross-sections of stacked fiber mats might be evaluated but the different materials involved (clot and air in the blood compartment of the ML vs. gas filled rigid PMP and TPU fibers) so far did not permit representative transverse sections.

## 5 Conclusion

All three imaging methods were capable of detecting clots within MLs, Thus, each of them showed specific advantages and disadvantages. MDCT was particularly suitable for a rapid estimation of the occurrence and distribution of clot structures in MLs. The high-resolution μCT allowed the detection of very small clot structures on individual gas fibers. Further, the visualization of smallest deviations of the fiber structure within the ML was possible. However, both CT-based methods required expensive analysis equipment and experienced staff. In contrast, FMI was a simple, cost-efficient, and straightforward method for localization of clots and estimation of their volumes. During preparation for FMI, the ML had to be disassembled, leading to a loss of the 3D clot structure, but the localization and intensity of the red clots could still be determined. Furthermore, FMI preparation facilitated the histological analysis. Clot-free areas were covered with evenly distributed leukocytes and PLAs proving the existence of thrombo-inflammatory processes in response to foreign body material. Of particular interest may be PLAs as blood markers for non-invasive monitoring of clot formation in ECMO systems. The occurrence of vWF cobwebs indicated the presence of elevated shear rates in MLs. Thus, shear induced clot formation seems more complex and remains a black box. The findings need to be extended to more MLs to identify common clotting mechanisms.

## Data availability statement

The raw data supporting the conclusions of this article will be made available by the authors, without undue reservation.

## Ethics statement

The studies involving humans were approved by Ethics Committee of the University Regensburg. The studies were conducted in accordance with the local legislation and institutional requirements. The human samples used in this study were acquired from blood clots retrieved from clinically used ECMO membrane lungs. Written informed consent for participation was not required from the participants or the participants’ legal guardians/next of kin in accordance with the national legislation and institutional requirements.

## Author contributions

MW: Conceptualization, Formal analysis, Investigation, Methodology, Validation, Writing – original draft, Writing – review & editing. MK: Conceptualization, Investigation, Methodology, Software, Validation, Visualization, Writing – original draft, Writing – review & editing. LK: Conceptualization, Supervision, Writing – review & editing, Funding acquisition, Resources. DP: Data curation, Methodology, Software, Writing – review & editing. MF: Supervision, Writing – review & editing, Data curation. ML: Data curation, Resources, Supervision, Writing – review & editing. KL: Funding acquisition, Investigation, Methodology, Resources, Supervision, Writing – review & editing.
