# Supplementary material for: Computer based visualization of clot structures in extracorporeal membrane oxygenation and histological clot investigations for understanding thrombosis in membrane lungs
Source: Front Med (Lausanne). 2024 Jun 19;11:1416319. doi: 10.3389/fmed.2024.1416319 (PMC11219572; doi:10.3389/fmed.2024.1416319)
Supplement: Supplementary file 1 [file Data_Sheet_1.PDF]

Supplementary Material

Computer Based Visualization of Clot Structures in Extracorporeal Membrane Oxygenation and Histological Clot Investigations for Understanding Thrombosis in Membrane Lungs

Maria S. Wagner[1] †, Michael Kranz[2,3] †, Lars Krenkel[2,3], Daniel Pointner[2,3], Maik Foltan[1], Matthias Lubnow[4], and Karla Lehle[1*]

*** Correspondence:** Karla Lehle: Karla.Lehle@ukr.de

1 Supplementary Data

1.1 Patient data

The study analyzed the clot burden of the final oxygenator from one patient. Selected patient data were listed in tab. S1.

1.2 Fiber mat image evaluation (FMI)

The ML housing was disassembled, the internal gas fiber stack was removed, and the upper left corner of each fiber mat was cut off to facilitate maintaining the fiber mat orientation (supplementary Figure S1 A). The single fiber mats were placed on a 3D-printed frame with the inlet side facing upwards. That frame had an edge length 10 mm smaller than the fiber mats to ensure a secure hold during photography. The resulting ROI was thus smaller than with the µCT or MDCT. A Nikon D3400 camera [Nikon, Shiyoda, Tokyo, Japan] and a ring flash [Walimex pro TTL MACRO Ringblitz, Altena, Germany] were used. With camera settings: Distance to fiber mat 25 cm, focal length 24 mm, shutter speed 1/160, aperture 16, photo sensitivity ISO 400, resolution 6000 x 4000 pixels. Ring flash settings: Multimode, power control 1/64, frequency 2 Hz.

After the image of the front side was taken, the fiber mat was rotated horizontally, and an image of the back side was taken. The process was repeated for all 119 fiber mats. All images were converted from raw NEF format to an 8-bit TIF file using Capture NX-D software [Nikon, Shiyoda, Tokyo, Japan]. Further image processing was carried out with an in-house developed Python 3.10 algorithm. This algorithm cropped the images to the size of the ROI (3182 x 3182 pixels) and converted them to binary images (threshold 160; result binarization process in supplementary fig. S1 B: Black (clotted) pixels and white (clot-free) pixels were counted). Proportion of black pixels was multiplied with the area of the fiber mat and half of the fiber diameter to receive the clot volume per image. This was repeated for all front and back sides of the fiber mat layers. The total clot volume was calculated from the sum of all clots of the individual fiber mats. The fiber mat removal process during preparation left large portions of the clots on the front side of the underlying fiber mat. In contrast to the gas fibers, the heat exchanger fibers showed a higher transparency. This had the consequence that clot structures on the front side of the heat exchanger fibers were additionally detected on the back side during photography. Using three randomly selected heat exchanger fiber mats, the back side was visually evaluated for clot structures and an adjustment factor of 0.01 was determined. This means that only one percent of the black pixels detected were considered blood clots on the back surface.

1.3 Immunofluorescence staining and microscopic methods

Selected gas exchange fiber mats were separately washed with phosphate-buffered saline (PBS) before cutting samples (0.5 cm x 1.0 cm) with a scalpel. Samples were washed in tris-buffered saline (TBS; 3x 5 min, room temperature, RT) and in TBST 0,1 % (TBS + 0.050 % Triton X100 + 0.050 % Tween 20) [Sigma, Madison, USA] (1x10 min, RT), blocked with blocking buffer (TBST 0,1 % + 2% normal donkey serum + 0.2 % cold water fish gelat,120 min, RT), and incubated with (1) a monoclonal mouse, anti-human CD42b [1:200; Novus, Gudensberg, Germany] and a polyclonal rabbit, anit-human vWF [1:500; Dako, Wiesentheid, Germany] antibody, or (2) a monoclonal rabbit, anti-human CD42b [1:200; Sigma-Aldrich, Taufkirchen, Germany] and a monoclonal mouse, anti-human CD62P [1:200; P-Selectin, SantaCruz, Santa Cruz, USA] antibody or (3) a monoclonal mouse, anti-human fibrin [1:350, Merck Chemicals, Darmstadt, Germany] antibody (12 h, 4 °C, in blocking buffer). After washing in TBST 0,1 %, 0,05 % and 0,005 % (5 min each) and TBS (5x5 min, RT), the samples were stained with monoclonal secondary antibodies (donkey anti-rabbit IgG (1:600); donkey anti-mouse IgG (1:200) conjugated with AlexaFluor AF594 (red) or AF488 (green) [both Dianova, Hamburg, Germany]) (in TBS, 60 min, RT, darkness). After washing with TBS (4x, 5 min, RT, darkness), samples were mounted between two cover slips embedded in Fluoromount-G® + DAPI [ThermoFisher, Waltham, USA] (48 hours, 4°C).

For micrographs (1600 × 1200 pixels) a Leica DMRBE fluorescence microscope [Leica Microsystems, Wetzlar, Germany] and a Spot2000 camera [Diagnostic Instruments, Sterling Heights, MI, USA] under software control [Visiview®, Visitron Systems GmbH, Puchheim, Germany] were used. For each sample an overview was created (25-fold magnification). Further, 18 crossing points (CP) of adjacent gas capillaries (50-fold magnification) were subject to qualitative description of clotted samples (figs. 6 A2-F2,). In clot-free regions (figs. 6 A1-D1) 30 randomly selected positions were photographed (400x magnification) to get qualitative and quantitative data. Original grey scale micrographs were converted to RGB mode separately for each fluorescence channel and merged together for co-localization of displayed structures using Adobe Photoshop CS5 (64 bit) [Adobe Inc., San Jose, USA].

2 Supplementary Figures and Tables

2.1 Supplementary Tables

Table S1. Selected inflammation, coagulation, hemolysis and technical ECMO data of the study patient during ECMO support

ECMO time	0	1	2	3	4	5	6	7	8	9	10	11	12	13	14	Post
Oxygenator	Pre	PLS					Ex	PLS							End	Post
CRP	296	251	163	107	87	54	41	33	21	27	55	103	79	66	68	75
Leukocytes	14	12.9	14.5	12.6	11.1	11	12.4	11	10	9.6	11	9.1	8.8	8	7.4	6.2
IL-6	931	235	nd	nd	nd	18	nd	nd	nd	nd	122	nd	nd	nd	86	nd
PCT	17	12.7	7	nd	2	nd	nd	nd	nd	nd	0.4	nd	nd	nd	nd	nd
aPTT	43	74	69	52	41	42	42	45	48	48	48	56	60	60	78	47
Platelets	374	327	340	333	322	240	192	176	171	158	173	177	170	152	116	117
FG	659	593	496	491	456	405	396	368	373	398	457	546	520	489	389	387
DD	6	5	4	5	8	9	11	7	4	5	9	17	27	33	36	15
INR	1.2	1.5	1.5	1.3	1.3	1.4	1.3	1.2	1.2	1.2	1.2	1.2	1.2	1.3	1.3	1.2
LDH	435	382	372	340	399	389	399	351	315	305	293	314	276	322	393	275
fHb	48	24	34	31	17	27	11	45	33	44	44	58	36	48	52	32
Blood flow	0	3.2	3.7	3.6	3.3	3.3	2.9	3.1	3.1	2.7	2.5	2	2.2	2.2	1.5	0
Gas flow	0	6	8	8	8	9	**12**	6	6	6	6	6	7	8	5	0
dpML	0	nd	nd	17	nd	20	**46**	16	nd	nd	nd	12	20	10	20	0
CO2 transfer	0	240	244	263	191	208	**84**	198	180	246	210	148	141	158	54	0
O2 transfer	0	192	192	173	122	135	131	139	143	170	138	100	112	123	51	0

ECMO time / days including days before and after ECMO therapy; PLS, permanent life support; CRP, C-reactive protein / mg/l; leukocytes / 1000/µl; IL-6, interleukin-6 / ng/l; PCT, procalcitonin / ng/ml; aPTT, activated partial thrombin time / s; platelets / 1000/µl; FG, fibrinogen / mg/dl; DD, D-dimer / µg/l; INR, international normalized ratio; LDH, lactate dehydrogenase / U/l; fHb, plasma free hemoglobin / mg/l; blood/gas flow / l/min; dpML, pressure drop across the membrane lung / mmHg; CO2/O2-transfer / ml/min; nd, not detected; pre, data before ECMO implantation; ex, exchange of the

ECMO system; end; end of therapy (weaning); post, data at day one or two after end of therapy; gray fields, emphasis of important data.

2.2 Supplementary Figures

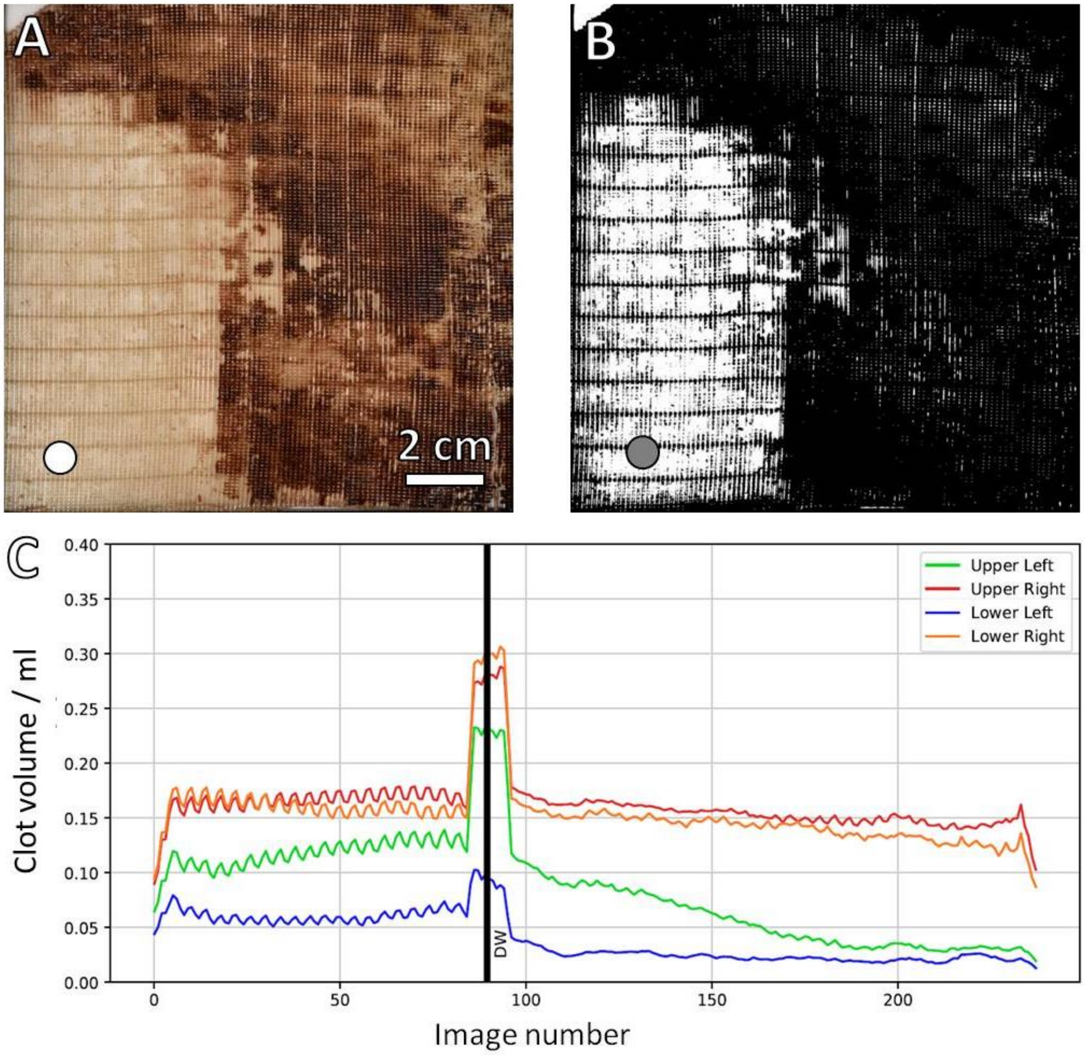

Supplementary Figure S1: FMI clot detection. (A) Raw image of fiber mat; bright areas are clot-free and dark areas are clotted. (B) Clots detected by algorithm; white areas are detected clot-free and black areas are detected as clots; (circles) position of inlet port. (C) Clot volume per image visualized with FMI for n = 119 fiber mats (238 images); for better readability, a convolution with a square kernel of size 7 was applied to each curve.

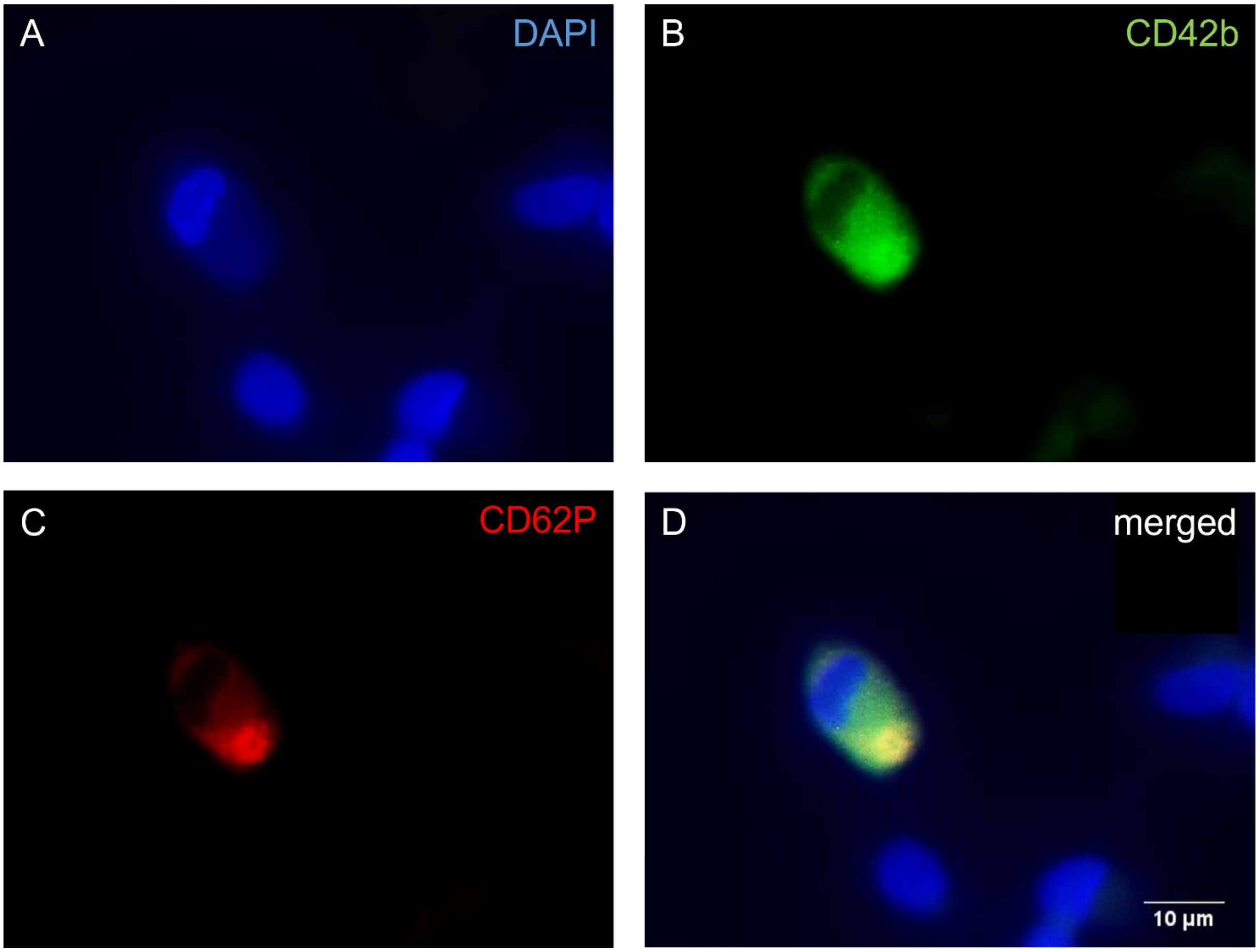

Supplementary Figure S2. P-selectin positive PLA. A platelet leukocyte aggregate (PLA) consisting of (A) a leukocyte (DAPI-stained nucleus) with (B) adherent CD42b-positive platelets. (C) In this case the platelets are mostly activated displaying P-selectin (CD62P) on their surface. (D) Merged image.
